# Supplementary material for: Improvement of Symptoms and Cardiac Magnetic Resonance Abnormalities in Patients with Post-Acute Sequelae of SARS-CoV-2 Cardiovascular Syndrome (PASC-CVS) after Guideline-Oriented Therapy
Source: Biomedicines. 2023 Dec 14;11(12):3312. doi: 10.3390/biomedicines11123312 (PMC10742066; doi:10.3390/biomedicines11123312)
Supplement: Supplementary file 1 [file biomedicines-11-03312-s001.zip › biomedicines-2742243-supplementary.pdf]

Improvement of Symptoms and Cardiac Magnetic Resonance Abnormalities in Patients with Post-Acute Sequelae of SARS-CoV-2 Cardiovascular Syndrome (PASC-CVS) after Guideline-Oriented Therapy

Mariann Gyöngyösi [1,*], Ena Hasimbegovic [1], Emilie Han [1], Katrin Zlabinger [1], Andreas Spannbauer [1], Martin Riesenhuber [1], Kevin Hamzaraj [1], Jutta Bergler-Klein [1], Christian Hengstenberg [1], Andreas Kammerlander [1], Stefan Kastl [1], Christian Loewe [2] and Dietrich Beitzke [2]

Supplementary Table S1. Detailed CMR data in the patients with/without CMR abnormalities and the data of the CMR phenotype groups.

	Cardiac abnormalities		Subtypes n and percentages of the CMR abnormalities within groups		
	No	Yes	Isolated pericardial effusion	Functional and/or morphological cardiac impairment	Combined
	n=123	n=155	34 (21.9%)	79 (51.0%)	42 (27.1%)
Age (years)	44±14	43±12	48±12 +	42±11	42±14
Female gender	99 (80.5%)	97 (62.6%)	27 (79.4%)	41 (51.9%)	29 (69.0%)
Pericardial effusion (w/wo functional impairment) n (%)	0	72 (46.5%)	34 (100%)	0	38 (90.5%)
Reduced LVF n (%)	0	39 (25.2%)	0	22 (27.8%)	17 (40.5%)
Reduced RVF n (%)	0	55 (35.5%)	0	37 (46.8%)	18 (42.9%)
Biventricular enlargement n (%)	0	21 (13.5%)	0	14 (17.7%)	7 (16.7%)
Isolated LV enlargement n (%)	0	56 (36.1%)	0	38 (48.1%)	18 (42.9%)
Isolated RV enlargement n (%)	0	47 (30.3%)	0	33 (41.8%)	14 (33.3%)
Myocardial edema n (%)	0	9 (5.8%)	0	5 (6.3%)	4 (9.5%)
T1 increase n (%)	0	5 (3.2%)	0	4 (5.1%)	1 (2.4%)
Non-ischemic late gadolinium enhancement n (%)	0	35 (22.6%)	0	17 (21.5%)	18 (42.9%)
Pleural effusion n (%)	3 (2.4%)	13 (8.4%)*	5 (14.7%)	3 (3.8%)	5 (11.9%)
Pericardial effusion (mL)	0	4.6±3.2*	4.2±2.3	0	5.1±3.8
CMR LV EF (%)	60±5 %	58±7*	64±7	57±6	54±7
CMR LV EDV (mL)	128±25	152±40*	125±24	162±43	153±35
CMR LV ESV (mL)	52±14	65±22*	47±15	71±22	69±18
CMR RV EF (%)	58±5	53±6*	58±5	51±5	51±7

CMR RV EDV (mL)	133±24	164±42*	131±27	173±44	168±37
CMR RV ESV (mL)	56±13	80±26*	56±13	87±24	82±27

Abbreviations: LV, left ventricular; RV, right ventricular; LVF, left ventricular function; RVF, right ventricular function; EF, ejection fraction; EDV, end-diastolic volume; ESV, end-systolic volume.

*p<0.01 between patient groups with/without CMR abnormalities. Parameter with zero value in one group was not included into the statistics.
+p<0.05 between the subgroups
